# What influences the effective practice and role optimisation of specialist/advanced paramedics working in emergency departments? A qualitative study

**DOI:** 10.29045/14784726.2019.06.4.1.40

**Published:** 2019-06-01

**Authors:** Alan Clarke

**Affiliations:** University of Wolverhampton

## Abstract

**Aims:**

Little is known about the experiences of paramedics who have left the ambulance service to work in emergency departments (ED). This study sought to explore the lived experiences of paramedics working in specialist/advanced ED roles, focusing on role transition, influences on effective clinical practice and perceptions of role optimisation. A secondary aim of the study was to make recommendations on the future development of specialist/advanced ED roles for paramedics.

**Methods:**

This was a qualitative study utilising descriptive phenomenology to collect and describe the lived experiences of participants via semi-structured interviews. Purposive and convenience sampling identified three emergency care practitioners (ECP), three student ECPs and two advanced clinical practitioners working across five EDs to participate in the study; all were Health and Care Professions Council (HCPC) registered paramedics. Interview data were transcribed verbatim and analysed using inductive thematic analysis.

**Results:**

This research produced a number of key findings:

- Transition to the ED involves significant adjustment to a new clinical environment, and new responsibilities and decision making, which can lead to a perception of regression to a novice practitioner.
- Pre-hospital assessment and history taking skills, and experience of autonomous working are pertinent enablers to effective practice within the ED.
- Support and mentorship from ED colleagues is available to enhance practice development.
- A limited access to medicines emerged as a significant barrier to daily practice, which could affect the patient experience. This also contributed to perceptions of sub-optimal working for many participants.
- Misconceptions over paramedic competencies could lead to role confusion and make inter-professional working difficult.
- Opportunities exist for future role expansion into areas such as resuscitation, majors and paediatrics within the ED environment.

**Conclusion:**

While role transition to the ED represents a turbulent period for paramedics, elements of pre-hospital paramedic practice transfer directly into new roles and contribute to effective practice. The paramedics in this study found that they were accepted and supported to work in the ED setting and spoke positively of expanding their roles into other areas of the ED in the future. A significant barrier to current clinical practice emerges from a lack of access to medicines, which impacts directly on the patient experience. The change in legislation to allow independent prescribing for advanced paramedics will address some of these issues, but interim improvements are required to extend existing arrangements to include paramedics; ultimately this will improve the quality and safety of care they are able to provide and the patient experience.

